# Quantitative analysis of facial symmetry by using three-dimensional technology

**DOI:** 10.1186/s12903-022-02315-x

**Published:** 2022-07-07

**Authors:** Zhouqiang Wu, Xiaolei Gao, Hu Long, Wenli Lai

**Affiliations:** State Key Laboratory of Oral Diseases and National Clinical Research Center for Oral Diseases and Department of Orthodontics, West China Hospital of Stomatology, Sichuan University, No. 14, Section 3, Ren Min South Road, Chengdu, 610041 Sichuan China

**Keywords:** Facial asymmetry, Three-dimensional, Subjective evaluation

## Abstract

**Background:**

Facial symmetry is becoming increasingly important in today's orthodontic treatment. But the asymmetrical boundary is not clearly demarcated. Stereophotogrammetry has a clear advantage in measuring facial asymmetry. The aim of this study was to quantify the facial asymmetry by three-dimensional (3D) technology as well as to study whether the evaluation by non-experts about facial asymmetry was consistent with the analysis by 3D technology.

**Methods:**

The facial symmetry of 330 patients was evaluated by 10 non-experts. 3D facial images were taken using 3dMD stereophotogrammetry equipment. The original face and its mirror shell were divided into 7 regions and the surface matching was measured in the whole face and all regional areas. The degree of symmetry was calculated by the software 3-matic STL 9.0. The difference between the two groups was analyzed by Independent-Samples T Test and the diagnostic efficiency of symmetry degree was analyzed by ROC curve analysis. The consistency between the symmetric degree and the result of evaluation was analyzed by Pearson correlation analysis.

**Results:**

The ROC analysis revealed significant diagnostic values in the determination of the facial asymmetry of lip, chin, cheek and lateral mandible areas. The cut-off values of symmetry degree were between 60 and 80%. The evaluation was middle correlation with the symmetric degree of the whole face.

**Conclusions:**

The chin and lateral mandible contribute most significantly to the facial symmetry. The objective measurement of facial symmetry, 3D technology, is reliable.

## Background

Facial symmetry is an indicator of good physical and genetic conditions [1, 2], and it is always pursued by both patients and clinician in our clinical practice. In contrast, facial asymmetry is indicative of an abnormal hyperplasia or resorption of the craniofacial bone, innate or acquired, and adversely affects people’s facial esthetics.

The absolute facial symmetry does not exist, while measurable facial asymmetry is quite widespread [3–5]. Although facial asymmetry compromises facial attractiveness, mild degree of asymmetry is often acceptable. Besides, the subjective evaluation and the objective measurement of the facial asymmetry have been realized by using various procedures and analyzed in many ways, from radiographic evidence [4] and anthropometric evidence [5] to a variety of values, (transverse linear and angular measurement [6] or triangulation [7], the midline to left and right difference used in different ways [8], and area super-impositions [9] on the postero-anterior (PA) cephalogram), from submento-vertex radiographs [10] to photographic techniques [11]. However, the borderline between acceptable asymmetry and significant asymmetry is still poorly understood and varies between professionals and laypersons [12]. The evaluation of facial symmetry is often subjective and has not been defined [13]. Orthodontists possess a certain expertise in assessing facial symmetry compared with both non-experts and general dentists [14]. Although orthodontists play a dominant role in evaluating patients’ facial symmetry, the assessment by non-experts living around orthodontic patients become more and more important in orthodontic diagnosis nowadays. Thus, non-experts’ decision is indispensable for the patients’ attitude towards facial symmetry.

Moreover, although facial symmetry depends mainly on skeletal tissues, the soft tissues are the ones building the facial contours and finally determining facial symmetry [13, 15, 16]. It is still controversial whether different facial regions contribute differently to the determination of facial symmetry [13, 15, 16]. Nevertheless, these studies evaluate facial symmetry based on 2D photographs, rather than 3D images and failed to incorporate non-experts’ views. Among all the 3D techniques, the 3dMD system was chosen for our study because it was more accurate and reproducible [17, 18].

Therefore, the aim of this study was to quantify the facial asymmetry by 3D technology as well as to study whether the subjective evaluation by non-experts about facial asymmetry was consistent with the analysis by 3D technology.

## Material and methods

### Participants

330 patients (185 males and 145 females) from West China Hospital of Stomatology, Sichuan University, Chengdu, China were included in this study. 3D facial images of all the patients were taken from September 2015 to December 2016 using a 3dMD stereophotogrammetry instrument (3dMD face TM System, 3dMD LLC, Atlanta, Georgia, USA) placing the participants in a natural head position. The inclusion criteria were as follows: both genders, aged 19–29 years, no history of orthodontic treatments. People with congenital craniofacial anomalies, severe facial deformities, severe malposition of eyes and ears were excluded.

### Ethics approval and consent to participate

This study was approved by the Ethical Committee of West China Hospital of Stomatology, Sichuan University (WCHSIRB-D-2018-072). Written informed consent was obtained from all the participants above 16 years old, or from both the participants and their parents for those under 16 years old.

### 3D facial image acquisition

**Fig. 1:**
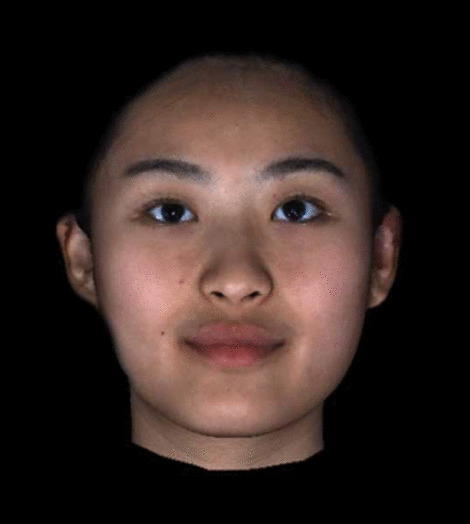
3D images

**Fig. 2:**
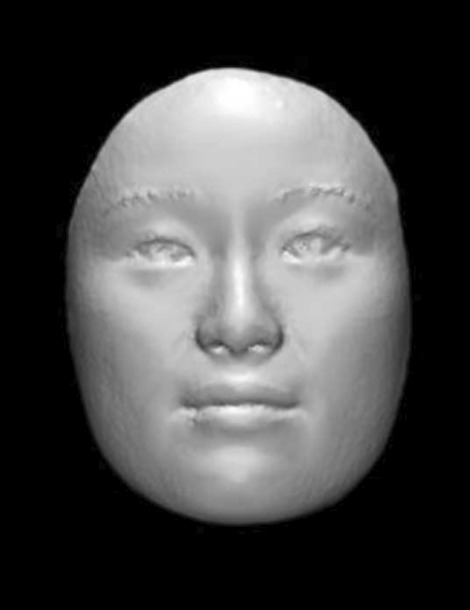
Removal of confounding regions such as ears, hair and neck

The 3D facial images (Figs. 1, 2) were taken under standardized conditions using the 3dMD stereophotogrammetry instrument. Patients were seated in front of the flash lamp and they looked at themselves in a mirror 5 feet away, thus ensuring that they were in a natural head position. The patients were informed regarding the face posture to assume: the expression should be relaxed, the mouth closed, and the facial muscles not contracted. Two sets of cameras were placed in front of the patient at the head level.

### Subjective evaluation of facial asymmetry

The evaluation committee consisted of 10 judges (5 males and 5 females, ranging 20–26 years) who did not receive any training on esthetic evaluation and did not know any participant. They were invited to evaluate whether a participant’s face was symmetric or asymmetric based on 3D facial images (Fig. 1). Each 3D image was shown to each evaluator for 30 s, without the possibility to look at the same image again. The images which obtained more than seven (7/10) evaluators’ agreement on symmetry or asymmetry were chosen to be measured in objective measurement. The inter-rater reliability of the 10 evaluators was assessed through modified Cohen’s kappa statistic [19].

### Objective measurement of facial asymmetry

**Fig. 3:**
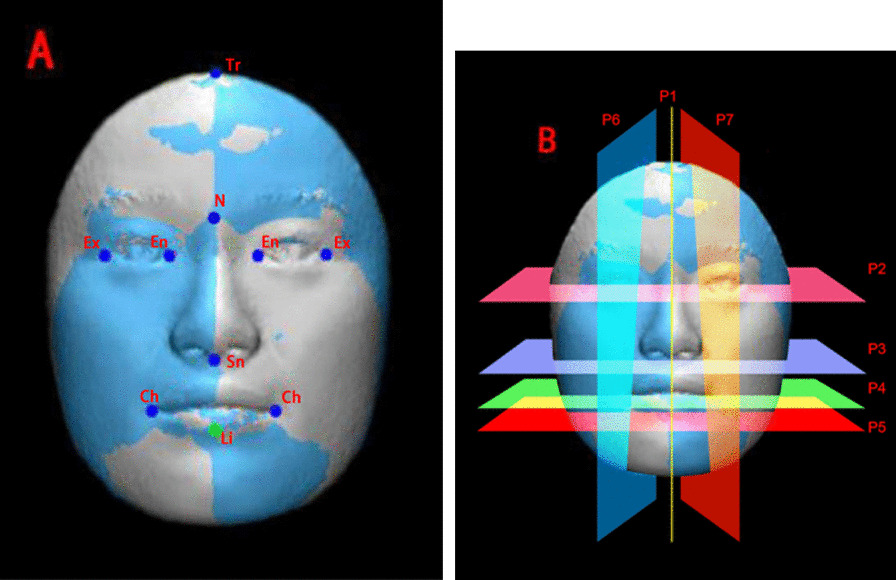
**A** Soft-tissue landmarks. Tr (Trichion): The point located below the hairline in the midline of the forehead. En (Endocanthion): The soft tissue point located at the inner commissure of each eye fissure. Ex (Exicanthion): The soft tissure point located at the outer commissure of each eye fissure. N: The nasion of soft tissue. Sn (Subnasale): The midpoint on the nasolabial soft tissure contour between the colunella crest and the upper lip. Ch (Cheilion): The point located at each labial commissure. Li (Labiale inferius): The midpoint of the vermilion line of the lower lip. **B** Constructed planes. P1 (The mirror symmetry plane): The plane across the points of Tr, N, Sn, Li. P2 (The transversal plane): The plane through left and right En and Ex. P3 (Subnasale): Through Sn, parallel to P2. P4 (Mouth corners): Through left Ch, parallel to P2. P5 (Lower mouth): Through Li, parallel to P2. P6 (Vertical left): Through left En and left Ch. P7 (Vertical right): Through right En and right Ch

**Fig. 4:**
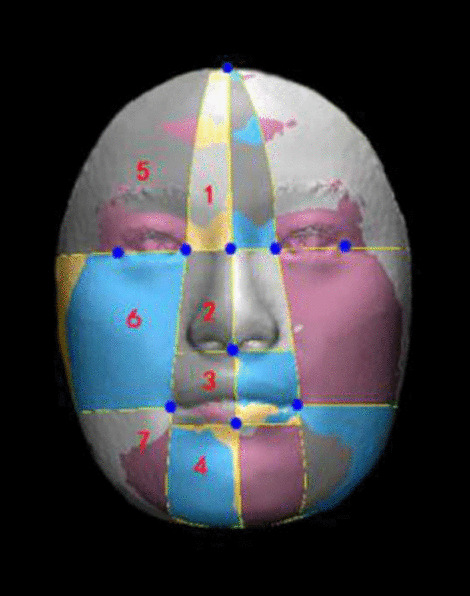
Regions for asymmetry analysis (1 = Central forehead, 2 = nose, 3 = lip, 4 = chin, 5 = lateral forehead, 6 = cheek, 7 = lateral mandible)

The objective evaluation of facial asymmetry included the whole face analysis and regional face analysis. The 3D facial images were imported into the 3-matic STL 9.0 (X64) and confounding factors (e.g., ears, neck and hair) were removed (Fig. 2). Then, soft tissue landmarks (Fig. 3A) and planes (Fig. 3B) were created on the images and the left and right side of face was divided into seven regions (Fig. 4), respectively: central forehead, nose, lip, chin, lateral forehead, cheek and lateral mandible. The mirror symmetry plane (P1, Fig. 3B), was mirroring performed on the whole and regional face. Besides, every region was performed separately.

**Fig. 5:**
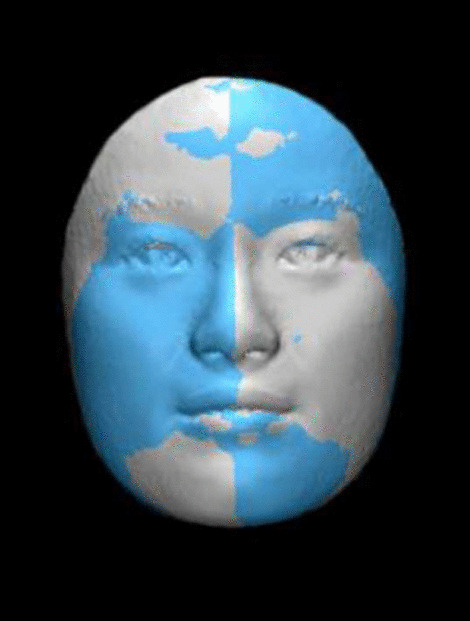
Superimposed image. The gray is initial image and the blue is mirrored shell

**Fig. 6:**
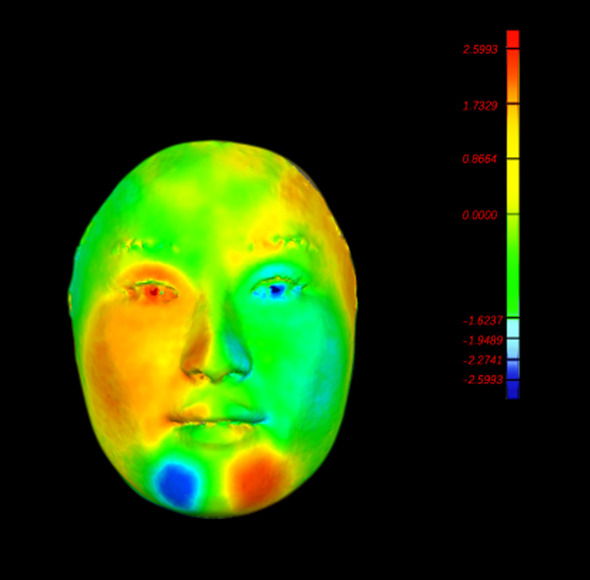
The color map matched the original and mirrored image

The whole face analysis, firstly obtained the mirror shell of left side face by the software (through the mirror image change). Then the mirror shell was superimposed with the right side face original image. Then the same process to analyze the right side face image (Fig. 5). After registration, shell-to-image deviations were graphically presented as color maps (Fig. 6) and quantitatively as histograms. This distance map illustrated the absolute distance between corresponding points on the original facial image and the mirrored shell.

The regional face analysis was firstly performed using the soft tissue reference points (Fig. 3A) which were established in previous studies [13, 15, 20, 21] to divide the whole face into 7 parts (Fig. 4). These landmarks were selected according to facial thirds and fifths that are important for the evaluation of facial symmetry. Then the same technique described above for the whole face analysis was used for the assessment of the regional face analysis. The color map of each specific region was also made.

Subsequently it was determined whether the distance between the two layers could be considered as symmetric. According to the past study, the relative distance between the point of the original image and the mirrored shell that was below 1 mm was superimposed and considered as symmetric. Otherwise, they were considered as asymmetric [22]. All the symmetric points constituted the symmetric areas and the asymmetric points form the asymmetric areas. Finally, the degree of symmetry for the whole face and each regional areas was showed by the percentage of symmetric areas.

### Statistical analysis

Statistical analysis was performed using the Statistical Package for Social Sciences Software version 18.0. The diagnostic efficiency of the regional degree of symmetry and the layperson subjective assessment were determined using the receiver operating characteristic (ROC) analysis. The consistency between the symmetric degree and the result of the evaluation was analyzed by the Pearson correlation analysis. A *p*-value less than 0.05 was considered statistically significant.

## Results

### Subjective evaluation results of facial symmetry

Initially, a total of 330 images were involved in this study. After the exclusion of the images with less than 70% agreement made by the 10 evaluators, 267 images (156 males and 111 females) were finally included in this study and the kappa value of the 10 evaluators is 0.7. Among the 267 subjects, 222 were considered as symmetric and the remaining 45 as asymmetric.

### The symmetry results calculated by the 3D software

**Fig. 7:**
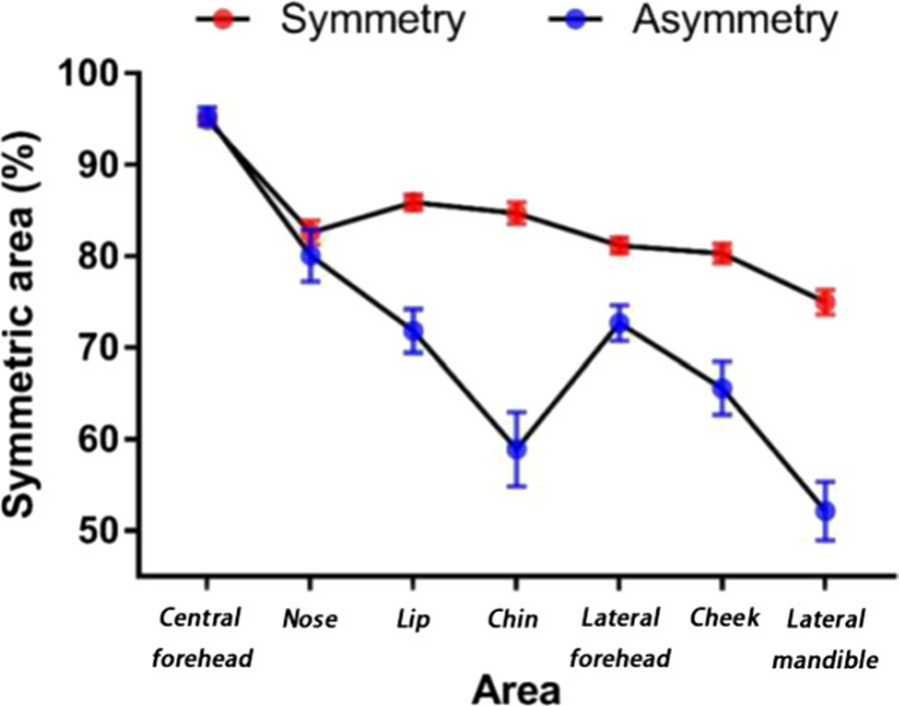
Line chart of degree of symmetry of different areas

All the data of the degree of symmetry of the whole face and the regional areas were normally distributed, as analyzed by the Independent-Samples T Test. The degree of symmetry for the whole face was significantly higher in the symmetric group (81.23 ± 10.66%) than in the asymmetric group (66.91 ± 13.17%) (*p* < 0.001) (Table 1 and Fig. 7). As regard the regional face analysis, the degree of symmetry of lip, chin, lateral forehead, cheek and lateral mandible was significantly higher in the symmetric group than in the asymmetric group (all *p* < 0.001).

**Table 1:** Degree of asymmetry of whole face and various facial regions

Facial region	Symmetry group^#^	Asymmetry group	*p* value
Whole face	81.23 ± 10.66	66.91 ± 13.17	< .001*
Central forehead	95.32 ± 6.67	93.74 ± 8.51	> .05*
Nose	82.33 ± 19.54	79.50 ± 21.65	> .05*
Lip	86.34 ± 12.15	75.72 ± 14.73	< .001*
Chin	85.24 ± 16.24	59.70 ± 27.11	< .001*
Lateral forehead	80.95 ± 12.64	74.68 ± 12.74	< .001*
Cheek	80.47 ± 15.35	66.77 ± 19.66	< .001*
Lateral mandible	74.30 ± 18.74	48.23 ± 24.53	< .001*

*Statistically significant difference (*p* < .05)

^#^Data are expressed as mean ± SD

**Fig. 8:**
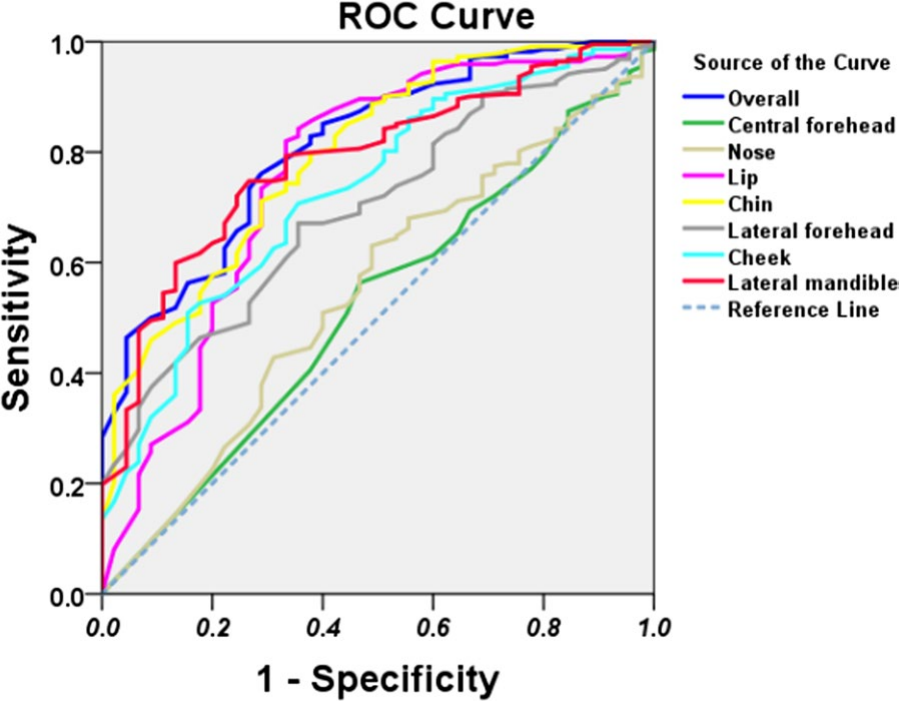
ROC analysis

The ROC analysis (Fig. 8) revealed a significant diagnostic value in the degree of symmetry of the specific regional areas, such as lip (AUC = 0.757), chin (AUC = 0.795), cheek (AUC = 0.729) and lateral mandible (AUC = 0.785), while no diagnostic value in the evaluation of symmetry was found considering the central forehead (AUC = 0.518), lateral forehead (AUC = 0.697) and nose (AUC = 0.547).

### The borderline between symmetry and asymmetry

The degree of symmetry of lip, chin, cheek and lateral mandible was significant in the diagnosis of facial symmetry. In order to obtain the cut-off value of the degree of symmetry in the diagnosis of facial symmetry, the cut-off value was calculated for each region when diagnosing the facial symmetry. According to the ROC analysis, the cut-off values were 75.5%, 79.5%, 74.5% and 64.5% for lip, chin, cheek and lateral mandible, respectively. For these cut-off values, the resulting sensitivity and specificity (symmetry was considered as a positive result while asymmetry was a negative result) were 0.82 and 0.67 for the lip, 0.71 and 0.71 for the chin, 0.71 and 0.64 for the cheek, and 0.75 and 0.73 for the lateral mandible. Moreover, the cut-off value for the overall facial region was 75.5%, with a sensitivity and specificity of 0.76 and 0.71.

## Discussion

It has been well documented that the ability of evaluating facial symmetry between orthodontists and non-experts is different. Orthodontists show better performance in assessing symmetry than laypersons [14]. Although the evaluation of orthodontic experts on facial symmetry is important for orthodontic diagnosis, non-experts also constitute the subgroup of people who daily assess the facial symmetry of patients. Thus, in order to get a more comprehensive diagnosis our orthodontists need non-experts’ evaluation about facial symmetry.

The points between the image and shell were regarded as symmetric points when the distance between those points was below 1 mm in our study. This threshold was set according to previous study where 1 mm was used as the threshold value for symmetry. Our results revealed that the inter-observer reliability of the 10 evaluators was substantial (*k* = 0.7), which indicating a good agreement among evaluators. This was in line with Edler’s and Masuoka’s studies, who previously reported kappa values of 0.68 and 0.77 for the agreement when evaluating the asymmetry group requiring treatment [15, 23].

Table 2 shows that the evaluation of facial symmetry is middle correlation with the symmetric degree of the whole face. As displayed in Table 1, the degree of symmetry of the whole face was significantly higher in the symmetric group than that in the asymmetric group (*p* < 0.001). The degrees of symmetry for different facial regions were examined in order to differentiate different facial regions in the contribution to facial symmetry. As displayed in Fig. 7, the degree of symmetry in the central forehead and nose did not differ between the symmetric and asymmetric group, while the degree of symmetry of lip, chin, lateral forehead, cheek and lateral mandible was significantly higher in the symmetric group than in the asymmetric one (all *p* < 0.001). The degree of symmetry of other facial regions was similar in the symmetric group except for the central forehead (symmetric area > 95%). However, the least symmetric regions in the asymmetric group were chin (60%) and lateral mandible (50%). These findings suggested that the central forehead and nose did not contribute to facial symmetry while chin and lateral mandible did. This is in disagreement with the results reported in previous studies [24], where the nose resulted an important factor in the determination of facial symmetry. Our hypothesis is that this disagreement might be due to the different types of enrolled subjects: cleft patients were included in COGHLAN’s [24] study. Our results were consistent with previous studies where the skeleton in the lower facial third is more asymmetrical than the upper one [7, 16, 25]. This may be due to the activity of the mobile temporomandibular joints and the contraction of the masticatory muscles, which are able to reshape the facial contours.

**Table 2:** Pearson correlation analysis between objective and subjective evaluation

	Symmetry	Overall
Symmetry		
Pearson correlation	1	− .459**
Sig. (2-tailed)		.000
N	267	267

**Correlation is significant at the 0.01 level (2-tailed)

The borderline between facial symmetry and asymmetry is obscure and difficult to define in clinical practice. Our further ROC analysis revealed that chin (AUC = 0.795), lateral mandible (AUC = 0.785), lip (AUC = 0.757) and cheek (AUC = 0.729) were significant in the diagnosis of facial symmetry. In contrast, central forehead (AUC = 0.518), lateral forehead (AUC = 0.697) and nose (AUC = 0.547) did not make much contribution to the diagnosis of facial symmetry. This result suggested that the lower facial third significantly contributed to the facial symmetry. Then, cut-off values of different facial regions were obtained to evaluate the facial symmetry, such as 75.5%, 79.5%, 74.5% and 64.5% for lip (sensitivity: 0.82; specificity: 0.67), chin (sensitivity: 0.71; specificity: 0.71), cheek (sensitivity: 0.71; specificity: 0.64) and lateral mandible (sensitivity: 0.75; specificity: 0.73), respectively. These results indicated that, when evaluate the regional facial symmetry, the use of the lip in the diagnosis of facial symmetry resulted in a facial symmetry diagnosed in those subjects with a degree of symmetry greater than 75.5%, while facial asymmetry was diagnosed in those with a degree less than 75.5%. Moreover, the cut-off value for the overall facial region was 75.5%, with a sensitivity and specificity of 0.76 and 0.71, with positive and negative predictive values of 0.93 and 0.34. This result suggests that, when we use this three-dimensional analysis for whole facial symmetry, we have 93% confidence with a positive result (facial symmetry) which indicate that the patients are symmetric and do not need treatment, while we are not confident with a negative result (facial asymmetry). Thus, this 3D technology could be used in helping clinic getting a more accurate diagnosis of facial symmetry.

Facial symmetry has been studied for several years. Past Studies on facial symmetry always depended on skeletal tissues. Such as the study [15, 16] which showed us the thresholds of the cephalometric indexes for subjective evaluation of facial asymmetry and the more important skeletal structure influencing the evaluation of facial symmetry in experts’ view. Obviously, these studies were extremely meaningful attempt to symmetric evaluation and had achieved significant results. Although our study doesn’t combine the experts’ view and non-experts’ view together, we found out that non-experts relied more on the lower facial thirds than the upper thirds when assessing the symmetry of the face as well as testing the 3D technology measurement. These may be contribute to the study about facial asymmetry in the future.

## Conclusion

In conclusion, the analysis of the facial symmetry by 3D technology, and subjective evaluation can be consistent in evaluating the facial asymmetry. The chin and lateral mandible contribute most significantly to the facial symmetry and that the lower facial third is the most asymmetric part.

## Data Availability

The datasets generated and/or analysed during the current study are not publicly available due [The clinical data of all participants in this study belongs to West China Hospital of Stomatology, Sichuan University and we need to obtain the approval of the hospital's medical department when obtaining it] but are available from the corresponding author on reasonable request.

## Abbreviations

2D: Two-dimensional
3D: Three-dimensional
